# DOPE/CHEMS-Based EGFR-Targeted Immunoliposomes for Docetaxel Delivery: Formulation Development, Physicochemical Characterization and Biological Evaluation on Prostate Cancer Cells

**DOI:** 10.3390/pharmaceutics15030915

**Published:** 2023-03-11

**Authors:** Thais da Silva Moreira, Alan Denis Olivindo Silva, Bianca Rodrigues Farias Vasconcelos, Elias da Silva Santos, Ana Carolina Cruz de Sousa, João Vito Barroso de Freitas, Yara Santiago de Oliveira, Laura Maria Teodorio Vidal, Fábio de Oliveira Silva Ribeiro, Alyne Rodrigues de Araújo, José de Brito Vieira Neto, Cláudia do Ó Pessoa, Raquel Petrilli, Josimar O. Eloy

**Affiliations:** 1 Department of Pharmacy, Faculty of Pharmacy, Dentistry and Nursing, Federal University of Ceará, Fortaleza 60430-355, CE, Brazil; 2 Institute of Health Sciences, University of International Integration of the Afro-Brazilian Lusophony- UNILAB, Redenção 62790-970, CE, Brazil; 3 Research Center on Biodiversity and Biotechnology (BIOTEC), Parnaíba Delta Federal University, Parnaíba 64202-020, PI, Brazil; 4 Department of Physiology and Pharmacology, Faculty of Medicine, Federal University of Ceará, Fortaleza 60430-275, CE, Brazil

**Keywords:** docetaxel, liposomes, factorial design, immunoliposomes, cetuximab, prostate cancer

## Abstract

Docetaxel (DTX) is a non-selective antineoplastic agent with low solubility and a series of side effects. The technology of pH-sensitive and anti-epidermal growth factor receptor (anti-EGFR) immunoliposomes aims to increase the selective delivery of the drug in the acidic tumor environment to cells with EFGR overexpression. Thus, the study aimed to develop pH-sensitive liposomes based on DOPE (dioleoylphosphatidylethanolamine) and CHEMS (cholesteryl hemisuccinate), using a Box–Behnken factorial design. Furthermore, we aimed to conjugate the monoclonal antibody cetuximab onto liposomal surface, as well as to thoroughly characterize the nanosystems and evaluate them on prostate cancer cells. The liposomes prepared by hydration of the lipid film and optimized by the Box–Behnken factorial design showed a particle size of 107.2 ± 2.9 nm, a PDI of 0.213 ± 0.005, zeta potential of −21.9 ± 1.8 mV and an encapsulation efficiency of 88.65 ± 20.3%. Together, FTIR, DSC and DRX characterization demonstrated that the drug was properly encapsulated, with reduced drug crystallinity. Drug release was higher in acidic pH. The liposome conjugation with the anti-EGFR antibody cetuximab preserved the physicochemical characteristics and was successful. The liposome containing DTX reached an IC_50_ at a concentration of 65.74 nM in the PC3 cell line and 28.28 nM in the DU145 cell line. Immunoliposome, in turn, for PC3 cells reached an IC_50_ of 152.1 nM, and for the DU145 cell line, 12.60 nM, a considerable enhancement of cytotoxicity for the EGFR-positive cell line. Finally, the immunoliposome internalization was faster and greater than that of liposome in the DU145 cell line, with a higher EGFR overexpression. Thus, based on these results, it was possible to obtain a formulation with adequate characteristics of nanometric size, a high encapsulation of DTX and liposomes and particularly immunoliposomes containing DTX, which caused, as expected, a reduction in the viability of prostate cells, with high cellular internalization in EGFR overexpressing cells.

## 1. Introduction

Prostate cancer is a serious health problem with a worldwide prevalence rate of 1,193,715 in 2020 [1]. Docetaxel (DTX) is the gold standard drug used in the treatment of patients with metastatic castration-resistant prostate cancer (mCRPC) [2]. DTX is a drug of the taxane class, a semi-synthetic derivative of 10-deacetylbaccatin III, and the mechanism of action occurs when the drug binds to cellular microtubules, leading to their assembly and subsequent stabilization, thus inhibiting their depolymerization and blocking normal cell division [3]. Despite the excellent efficacy results of DTX in the treatment of mCPRC, with reduced PSA levels and increased patient survival, clinical studies demonstrated a marked toxicity profile with the onset of neutropenia, anemia, thrombocytopenia, neuropathy and gastrointestinal disorders [4].

Concerning the side effects related to taxotere^®^ (commercial drug), nanotechnology is a viable alternative to promote cancer selective delivery, reduce side effects and improve treatment efficacy. Thus, among the various types of nanosystems, liposomes, nanospheres with concentric lipid bilayers formed of phospholipids—which can be natural or synthetic—and biocompatible, biodegradable and non-immunogenic materials, stand out for the ability to load hydrophilic and lipophilic drugs. In order to increase the circulation half-life, pegylation is commonly used, since PEG forms a steric impediment in the external part of the vesicle and prevents recognition by reticulo-endothelial system [5,6,7]. 

For complex formulations development, such as liposomes, achieving optimal physicochemical parameters, reducing the number of tests and saving materials and time, the design of experiments (DoE) can be used. As a Quality by Design (QbD) tool, DoE has been widely used within pharmaceutical industry to ensure process and product improvements, as regulatory agencies such as the ICH and FDA have incorporated it into their guidelines (Q8-11). Among the advantages of QbD, one can highlight the ability to identify the variability of process parameters and attributes, leading to reduction of failures in the batch. Furthermore, QbD increases robustness and ensures that the method achieves the intended performance [8]. The Box–Behnken factorial design model, employed herein, combines high and low factor levels and their midpoints; thus, it is possible to formulate a list of experiments with combinations of the three levels and, based on the results obtained, carry out a statistical evaluation of the parameters to be achieved, in order to select an optimized formulation in the end [9]. In this scenario, Vardhan et al. (2017) developed polymeric nanoparticles containing docetaxel using Box–Behnken design, as well as Eloy et al. (2020), who developed immunoliposomes for DTX encapsulation. However, they employed a conventional lipid composition, based on soy phosphatidylcholine and cholesterol [10,11]. However, nanosystems able to specifically deliver DTX at the target, such as stimuli-responsive formulations, are still needed. Furthermore, the combination of pH-sensitive liposomes and EGFR targeting has not been investigated so far.

In order to rapidly divide, tumor cells stimulate the production of fenestrated blood vessels. The presence of fenestrations facilitates the extravasation and permeation of nanoparticles into tumors, where nanoparticles accumulate. This factor is known as the increased tumor permeability and retention (EPR) effect, which contributes to the retention of nanoparticles in the tumor environment and delivery of the drug to exert a cytotoxic effect. To improve the selective delivery of encapsulated drugs, pH-sensitive liposomes can be developed. It is known that the tumor extracellular environment has a pH between 5.8 and 7.2, and within the endosomes of tumor cells the pH is 5.5. Therefore, pH-sensitive liposomes (formed with DOPE and CHEMS, for example) undergo structural changes under acidic stimulus, triggering the release of drugs into the cell cytoplasm [12]. 

In order to increase the tissue selectivity of treatment and increase the efficiency of drug release in the tumor environment, liposomes can be functionalized with monoclonal antibodies that have a high affinity for some overexpressed molecule on the cancer cell surface. Immunoliposome undergoes receptor-mediated internalization, in which the antibody binds to a protein on the cell surface that promotes the entry of immunoliposome into endosomes and the subsequent degradation of immunoliposome in the cell cytoplasm, leading to full drug release [13,14,15]. EGFR is a transmembrane tyrosine kinase receptor that is overexpressed in several cancers of epithelial origin, and its natural ligands include EGF and TGF-α. It has also been reported to be overexpressed on prostate cancer cells [16]. Cetuximab is a human–murine chimeric monoclonal antibody with a high affinity for EGFR (Kd: 0.1–0.2 nM). It stimulates antibody-dependent cellular cytotoxicity, provides receptor internalization and degradation and is an excellent active targeting strategy for liposomes carrying antineoplastic drug [17]. Therefore, we hypothesize herein that cetuximab-functionalized DOPE/CHEMS liposomes will enhance DTX delivery to metastatic prostate cancer cells, enhancing cytotoxicity, which has not been previously studied. 

Taking into account all the benefits of immunoliposomes in cancer treatment, the objective of this study is to develop, using a factorial design approach, pH-sensitive, anti-EGFR immunoliposomes for DTX delivery. We address formulation development, physicochemical characterization, liposomal functionalization with cetuximab and finally, cytotoxicity and cellular uptake on prostate cancer cells with low and high EGFR expression.

## 2. Materials and Methods

### 2.1. Preparation of pH-Sensitive Liposomes

The preparation of pH-responsive liposomes was based on the lipid film hydration method described by Bangham et al. (1965), in which lipids (DSPE PEG 2000 lipid (Lipoid, Germany) was fixed at 0.5 mol% and the DOPE:CHEMS (Lipoid, Germany and Sigma Aldrich Co., St. Louis, MO, USA, respectively) (molar ratios varied) and drugs (varying molar ratios, according to Table 1) were dissolved in 2 mL of chloroform in a round-bottom flask. Afterwards, the organic solvent was evaporated under reduced pressure in a rotary evaporator at a temperature of 60 °C, at 100 rpm for 30 min. Following evaporation, the lipid film was hydrated with 5 mL of PBS buffer (pH 7.4) under the same temperature for 30 min. Then, the formulation was subjected to sonication in probe ultrasound (Q500; QSonica, Newtown, USA) at 40% amplitude with 5/2 on/off cycles at variable times (2.5 min, 5 min or 10 min) [18,19].

#### 2.1.1. Box–Behnken Design

The Box–Behnken response surface design methodology was used to optimize liposome preparation. This experimental design is represented by a set of points located at midpoint of each edge and replicated central point of a multidimensional cube [20,21]. The number of experiments (N) required for the development of the Box–Behnken design was determined by *N =* 2*k* (*k −* 1) *+ C*_0_ (where *k* is the number of factors and *C*_0_ is the number of central points) [21]. A total of 15 experiments were performed at three levels: low, medium and high (−1, 0, +1), with three variables to choose the most appropriate composition formulation and test the correlation between dependent and independent variables, employing the minimum number of experiments. The central point was replicated three times. Formulation parameters and parameter levels were chosen based on preliminary studies [9,22]. The independent variables established were DOPE:CHEMS molar ratio (X_1_), lipid:DTX molar ratio (X_2_) and sonication time (X_3_). The dependent variables studied were encapsulation percentage (Y_1_), particle size (Y_2_) and polydispersity index (Y_3_). The ranges of formulation parameters and responses are shown in Table 1.

For each dependent variable, the influence of formulation factors (X_1_, X_2_ and X_3_) was represented using a quadratic model of second-order polynomial regression, generated by Minitab^®^ statistical software, expressed by the following Equation (1):

(1) $$Y_{i}=b_{0}+b_{1}X_{1}+b_{2}X_{2}+b_{3}X_{3}+b_{12}X_{1}X_{2}+b_{13}X_{1}X_{3}+b_{23}X_{2}X_{3}+b_{11}X_{1}^{2}+b_{22}X_{2}^{2}+b_{33}X_{3}^{3}$$

where Y_i_ represents a dependent variable, b_0_ is the intercept coefficient of the points, b_1_ to b_33_ are regression coefficients calculated from the observed experimental values, and X_1_, X_2_ and X_3_ are the independent variables selected before initial experiments. The terms X_1_X_2_, X_1_X_3_ and X_2_X_3_ represent the interaction effects, and the term X_i_^2^ (i = 1, 2 or 3) indicates the quadratic effects [8,23,24]. The result was statistically validated by analysis of variance, by statistical significance of coefficients and R^2^ values. Statistical analysis results were considered significant for *p*-values < 0.10.

#### 2.1.2. Functionalization of pH Sensitive Liposome with Cetuximab (Immunoliposome)

The method of liposome functionalization with cetuximab followed the direct conjugation described previously [11,25]. The cetuximab antibody (Merck Serono, Biberach, Germany) was diluted at different concentrations (2 mg/mL and 3 mg/mL) with a 5 mM PBS/EDTA buffer at pH 8.0 and a 50 mM PBS/EDTA buffer and, finally, the solution pH was adjusted to 8.0 with an NaOH solution (0.1 N). Traut’s reagent (Sigma Aldrich Co., St. Louis, MO, USA) (3 mg/mL) was added to the antibody solution, followed by incubation at 37 °C for 1 h. A thiolated antibody was purified by PD-10 column exclusion gel chromatography, collecting 14 fractions of 1 mL each, using a 5 mM PBS/EDTA buffer pH 8.0 as an eluent. Fractions were evaluated by the BCA (bicinchoninic acid) method, following the manufacturer’s protocol (Thermo Scientific, Pittsburg, PA, USA). In a 96-well microplate, 25 μL of each concentration of standard curve (25–750 μg/mL) and of the fractions obtained from the elution of the PD-10 column were added, then, in each well, 200 μL working reagent was added. The 96-well microplate was incubated at 37 °C for 30 min and the absorbance was read at 562 nm in a microplate reader (SpectraMax Plus 384, Molecular Devices, California, USA). Then, the fractions containing antibody were pooled and incubated for 2 h at 37 °C with previously prepared liposome, followed by overnight incubation at room temperature. To separate the liposome and unconjugated antibody, the immunoliposome was purified in CL-4B column (Sigma Aldrich Co., St. Louis, MO, USA) eluting with PBS pH 7.4 buffer, collecting 30 fractions of 1 mL each. In a 96-well microplate, 25 μL of each concentration of the standard curve (25–750 μg/mL) and 25 μL of the fractions obtained from the elution of the CL-4B column were added with 200 μL of working reagent. The microplate was incubated at 60 °C for 30 min and the absorbances were read at 562 nm in a microplate reader. In the chromatogram, the interference of lipids with a blank control liposome was discounted and the protein concentration of each fraction was obtained from BCA calibration curve. In order to compare and analyze the elution profiles, a 3 mg/mL antibody solution and liposome without DTX labeled with 1 mg of DIO fluorophore (Sigma Aldrich Co., St. Louis, MO, USA) were also purified alone on a CL-4B column, eluting with a PBS buffer, collecting 30 fractions of 1 mL each, following the same 96-well microplate procedures described above. The CE% of immunoliposome was calculated according to Equation (2) below.

(2) $$\mathrm{Conjugation}\;\mathrm{Effieciency}\;\left(\mathrm{EC}\%\right)=\frac{\mathrm{Protein}\;\mathrm{concentration}\;\mathrm{in}\;\mathrm{the}\;\mathrm{immunoliposome}\;\mathrm{fraction}}{\mathrm{Total}\;\mathrm{concentration}\;\mathrm{of}\;\mathrm{eluted}\;\mathrm{protein}}$$

### 2.2. Physicochemical Characterization of Liposomes and Immunoliposomes

#### 2.2.1. Particle Size, Polydispersity and Zeta Potential

Hydrodynamic size distribution, polydispersity (PDI) and zeta potential of the formulations were characterized using the Malvern Nanosizer ZS equipment (Malvern Instruments, UK). Size and polydispersity were evaluated by dynamic scattering of light at a wavelength of 633 nm. Measurements were performed using an optical laser operating at a detection angle of 173°. To determine the zeta potential, electrophoretic mobility of particles subjected to an electric field at 25 °C was evaluated, in which the particles migrate differentially to the oppositely charged electrode, with a velocity proportional to the magnitude of their charge. Before measurements, samples were diluted (1:10) in ultrapurified water and vortexed. All measurements were performed in triplicate and the results were expressed as mean ± standard deviation (SD).

#### 2.2.2. Encapsulation Efficiency

The determination of encapsulation efficiency was performed by spectrophotometry. For this, it was necessary to prepare a calibration curve. Initially, a stock solution of 1 mg/mL of docetaxel was prepared by dissolving the drug in acetonitrile (Sigma Aldrich Co., St. Louis, MO, USA) in an ultrasonic bath for 10 min. This solution was filtered through a polytetrafluoroethylene (PTFE) membrane with a pore size of 0.45μm. From the stock solution, solutions with concentrations of 1 μg/mL, 2 μg/mL, 5 μg/mL, 10 μg/mL, 15 μg/mL, 30 μg/mL and 50 μg/mL of docetaxel in acetonitrile were prepared in triplicate. Then, they were analyzed by UV spectrophotometry (U-2910, Hitachi, Japan) at a wavelength of 232 nm. All analyses were performed in triplicate. From these data, a curve was generated to correlate the absorbance values of encapsulation efficiency with the concentrations obtained. Encapsulation efficiency was performed by the direct method using the filtration technique. For analysis, the unloaded precipitated drug was removed by filtration through a 0.45 μm PVDF filter. In triplicate, purified and total liposomes were diluted (1:10) in acetonitrile, placed in an ultrasonic washer for 10 min, and then vortexed and filtered through a 0.45 μm PTFE filter [11]. After this treatment, the samples absorbance was determined by spectrophotometry at a wavelength of 232 nm. The percentage of encapsulation was calculated using the following Equation (3):

(3) $$\mathrm{EE}\;\left(\%\right)=\frac{\mathrm{Purified}\;\mathrm{liposome}\;\left(\mathrm{DTX}\right)}{\mathrm{Total}\;\mathrm{liposome}\;\left(\mathrm{DTX}\right)}\times 100$$

#### 2.2.3. Atomic Force Microscopy

Atomic force microscopy is a high-resolution technique/method that has the ability to demonstrate/illustrate the surface topology. The application of this methodology to immunoliposomes aims to demonstrate the presence of conjugated antibody on the liposome/nanoparticle surface. A total of 10 μL aliquot of each sample was deposited on a mica surface and allowed to stand for 15 min at 36 °C. TT-AFM equipment (AFM Workshop, South Carolina, USA) was used in intermittent contact mode (tapping mode), using TED PELLA silicon tips (TAP300-G10) with an amplitude frequency of 242.26 kHz. The Gwyddion 2.59 program was used to process the images, obtain the distribution profile by diameter, the mean diameter, the roughness distribution profile and the mean roughness of the liposomes. 

#### 2.2.4. Fourier Transform Infrared Spectroscopy (FTIR)

The drug and nanoparticle interaction was studied by subjecting the previously lyophilized samples to FT-IR spectroscopy in an FTIR spectrophotometer (IRTracer-100, Shimazdzu, Japan), with a horizontal attenuated total reflectance accessory. The scan was performed in the range of 4000 to 700 cm^−1^.

#### 2.2.5. Thermal Analysis

The curves of thermogravimetric (TGA) and differential scanning calorimetry (DSC) of free DTX and DTX-containing liposome and immunoliposome samples were recorded using a simultaneous thermal analysis equipment (Jupiter STA 449, Netzsch, Germany). The measurement was made from 30 to 400 °C using a heating rate of 10 K.min^−1^, and around 10 mg of sample was used in the analysis. The sample was placed in sealed aluminum crucibles with pierced lids, and during the experiment the flow of nitrogen (70 mL/min) was constant [26].

#### 2.2.6. Powder X-ray Diffractometry

The patterns of powder X-ray diffraction (PXRD) of free DTX and DTX- containing liposome and immunoliposome samples were obtained using a D8 Advanced system (Bruker AXS, Massachusetts, USA), equipped with a theta/theta goniometer, operating in the Bragg Brentano geometry, CuKα (0.15419 nm) radiation source and a LynxEye detector. The electric current and voltage applied were 40 mA and 40 kV, respectively. The opening slit for the beam incident on the sample was 0.6 mm and the samples were scanned within the scan range of 2θ = 5 to 40° [27].

#### 2.2.7. In Vitro Release Study

For the release study, the docetaxel liposome and docetaxel solution samples were diluted in 2 mL of phosphate buffer (pH 7.4) and in citrate buffer (pH 5.5) containing 1% sodium lauryl sulfate. Samples were put inside PVC tubes wrapped with 12–14 kDa MWCO (molecular weight cut-off) cellulose dialysis membranes and connected to the dissolution shafts of the apparatus 1, with agitation speed at 150 rpm and temperature at 37 °C, in 50 mL of buffer solution (receptor compartment). The samples were collected in the receptor compartment in different times, from 30 min to 72 h, with replacement with fresh medium at every time. Afterwards they were diluted, filtered (0.45 µm) and analyzed by the HPLC (L-2455U, Hitachi, Japan) method [28].

### 2.3. Antibody Functionalization Characterization

#### 2.3.1. Electrophoresis

The antibody presence after conjugation with the liposomes was assessed using SDS-Page gel electrophoresis under reducing conditions, based on previous studies by Petrilli et al. (2018). The samples at a concentration of 0.2 mg/mL were previously treated with the addition of a 2-mercaptoethanol (Sigma Aldrich Co., St. Louis, MO, USA) reducing reagent and Laemmli buffer, heated at 80 °C for 5 min and then applied to 15% SDS-Page gels. The electrophoretic run was conducted at 100 V for 1 h and 30 min using Tris/Glycine/SDS pH 8.3 buffer. Staining was performed using bromophenol blue dye (Synth, Brazil), followed by removal of excess dye with washes in distilled water. Finally, the gels were photographed to interpret the results [25].

#### 2.3.2. Thermophoresis

The thermal analysis and structural integrity of proteins was performed by the thermophoresis technique, through the emission of intrinsic fluorescence of tryptophan and tyrosine residues, in order to evaluate the protein denaturation and stability of biomolecules, generating information about changes in structure and conformation in a gradient temperature microscopic. The analysis was performed using the Tycho ™ NT equipment 6 (NanoTemper, München, Germany), where 10 µL of the samples were heated in silica capillaries from 35 **°**C to 95 **°**C. Data were analyzed using Nano-Temper Analysis software 1.3.2.880.

#### 2.3.3. Indirect ELISA

To evaluate the binding of the cetuximab antibody present on the surface of the liposome with the EGFR receptor, the indirect ELISA (enzyme-linked immunosorbent assay) method was performed. First, the EGFR antigen was diluted in 100 mM carbonate/bicarbonate buffer pH 9.6 to a final amount of 50 ng per well and then coated onto a microtiter plate at 4 °C overnight. After this period, the wells were washed three times with PBST buffer (PBS + 0.2% Tween) and then blocked for nonspecific binding with blocking solution (PBS + 5% skim milk) for 1 h at 37 °C. Then, the wells were washed again with PBST solution, therefore, the cetuximab (1 µg/mL), immunoliposome and liposome were incubated at the same concentration in blocking solution for 1 h at 37 °C. After this period, the wells were washed with PBST and the secondary antibody (Spring, Pleasanton, CA, USA) (anti-human IgG H&L (HRP)) was diluted in blocking solution at a ratio of 1:1000 and incubated for 2 h at 37 °C. The revealing substrate of the reaction is TMB (3,3′,5,5′ tetramethylbenzidine) which reacts with the enzyme HRP (horseradish peroxidase) present in the secondary antibody. Thus, after the incubation period, 90 µL of the TMB substrate solution was added to the wells and incubated for 15 min at 37 °C and then 50 µL of the 2 M H_2_SO_4_ stop solution was added. The plate was read in a microplate reader with absorbance at 450 nm. 

### 2.4. Cell Studies

PC3 (prostate carcinoma—low expression of EGFR) and DU145 (prostate carcinoma—high expression of EGFR), provided by the National Cancer Institute (USA) cells were cultured in RPMI 1640 M medium (Sigma Aldrich Co., St. Louis, MO, USA), supplemented with 10% Fetal Bovine Serum (FBS) (Sigma Aldrich Co., St. Louis, MO, USA) and 1% antibiotic/antimycotic solution (Sigma Aldrich Co., St. Louis, MO, USA), at 37° C with 5% CO_2_.

#### 2.4.1. Cellular Viability Assay

To assess cytotoxicity, MTT method was used, which is based on the activity of mitochondrial dehydrogenase enzymes, reducing MTT (yellow color) to formazan salt crystals (purple color). With the appropriate culture conditions, after reaching 90% confluence, the cells were trypsinized with 1:10 trypsin and seeded (25,000 cells per well) into a 96-well microplate and incubated for 24 h at 37 °C under the same conditions, recommended for each cell line, PC3 and DU145. After removing the complete culture medium, the experimental groups (pH sensitive liposome, pH sensitive immunoliposome, in addition to drug-free controls and positive controls, composed of DTX and cetuximab) were diluted in complete medium at the highest concentration of 1000 nM and added to the plates, which were incubated at 37 °C for 72 h. After the incubation period, the wells were washed with saline and complete medium with 3-[4,5-dimethyl-thiazol-2-yl]-2,5-diphenyltetrazolium bromide—MTT (Sigma Aldrich Co., St. Louis, MO, USA) (2.5 mg/mL), followed by incubation for 4 h at 37 °C. Then, the MTT-containing medium was discarded and DMSO added to the wells to dissolve the formazan salts. Absorbance was read at 570 nm [29]. To calculate the concentration resulting in 50% cell death (IC_50_), the optical density of the negative control (untreated cells) was marked as 100% and the result was calculated from the concentration-effect curves [30].

#### 2.4.2. Cellular Internalization

The uptake of liposomes and immunoliposomes (without drug, containing the fluorescent agent 3,3′-Dioctadecyloxacarbocyanine perchlorate, Dio, 0.5 mol%) was evaluated on DU145 and PC3 cells, respectively, with high and low EGFR expression by confocal microscopy and flow cytometry methods [31]. In the confocal microscopy method, cells were plated in 6-well microplates with sterile 22 mm/22 mm coverslips (5 × 10^5^ cells/well) and incubated for 24 h at 37 °C under the same conditions, which was recommended for each cell line, PC3 and DU145. Then, the wells were washed and the cells incubated with the samples diluted in incomplete medium for 24 h at 37 °C. After treatment, the wells were washed with saline and the cells fixed with 1% paraformaldehyde. After 15 min, the wells were washed again with saline and 4′,6-diamidino-2-phenylindole (DAPI) solution was added, which was left to rest for 10 min. Then, the wells were again washed with saline and the coverslips were poured onto histology slides containing Fluoromount. The slides were kept at rest under refrigeration, protected from light, prior to viewing under a confocal microscope (Zeiss 5 Live, Zeiss, Germany, λexc = 488 nm, λem = 552 nm, with a 20 X objective) [11,25]. For flow cytometry, 5 × 10^5^ cells/well were plated in 6-well microplates and incubated for 24 h at 37 °C under the same conditions mentioned. Then, the cells were washed and incubated with the samples diluted in incomplete medium for 4 and 24 h at 37 °C. The cells were trypsinized, centrifuged and resuspended in saline solution and then they were added with propidium iodide (Thermo Scientific, Pittsburg, PA, USA) (5 μL of 50 μg/mL solution) and submitted to analysis in flow cytometer (FACSCalibur, BD, New Jersey, USA), using λexc = 488 nm, λem = 530/30 nm, for Dio and λexc = 488 nm and λem = 670 nm for propidium iodide [11,25].

### 2.5. Statistical Analysis

The results of factorial design were analyzed by Minitab^®^ and Design-Expert^®^. The result was statistically validated by analysis of variance, by statistical significance of coefficients and R^2^ values. Statistical analyses were considered significant for *p*-values < 0.01. Atomic force microscopy, cell internalization and indirect ELISA results were analyzed using GraphPad Prism 8.0.1. Atomic force microscopy was expressed as mean ± Standard Error of Mean (SEM). A *p* < 0.05 was considered statistically significant. The result of cell internalization by cytometry was analyzed by a two-way ANOVA test with a Bonferroni posttest. A *p* < 0.01 was considered statistically significant. The indirect ELISA was analyzed by a one-way ANOVA test with a Tukey posttest between samples. A *p* < 0.05 was considered statistically significant.

## 3. Results and Discussion

Formulation composition and liposomal preparation protocol were chosen according to previous formulation studies carried out in our group and based on previous studies [9,32]. The composition based on DOPE and CHEMS takes into account their ability to form pH sensitive liposomes. This lipid combination is known for its fusogenic capacity, preferential release in the acidic tumor microenvironment and also for more efficient endosomal escape within cancer cells [33,34,35]. The fusogenic behavior of these nanoparticles is due to DOPE, a neutral lipid that, in an aqueous medium, tends to acquire an inverted hexagonal structure, not promoting the formation of lipid vesicles. Liposome formation with DOPE requires the addition of stabilizing agents, such as cholesteryl hemisuccinate (CHEMS), which, when ionized (at neutral or alkaline pH), stabilizes the lamellar form of DOPE for vesicle formation. However, when under acidic pH, the protonated or molecular form of CHEMS destabilizes the vesicular system, leading to encapsulated materials release [35,36,37,38].

**Figure 1:**
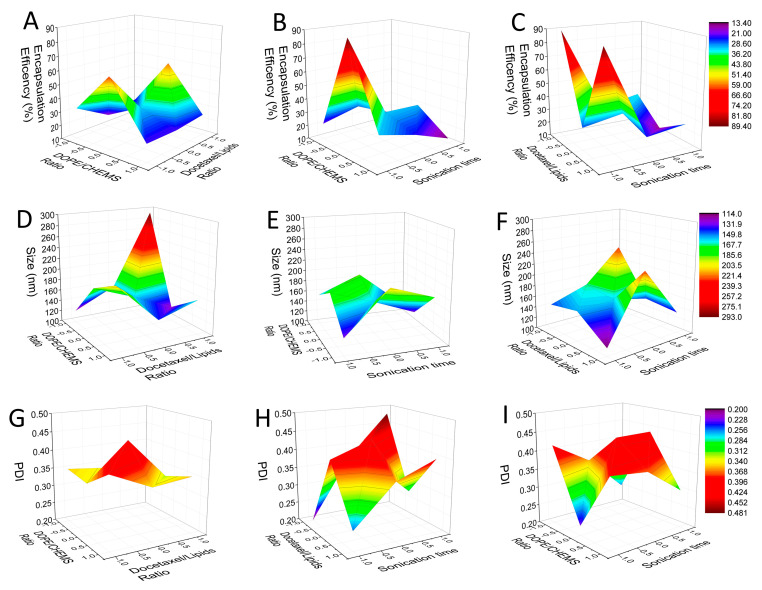
Response surface plots showing the effects of various formulation parameters on the responses encapsulation efficiency (Y_1_), particle size (Y_2_) and polydispersity index (Y_3_). The (**A**–**C**) response surface plots relate DOPE/CHEMS ratio (X_1_), docetaxel/lipid ratio (X_2_) and sonication time (X_3_) to encapsulation efficiency (Y_1_). The (**D**–**F**) plots to the effects of X_1_, X_2_ and X_3_ on particle size (Y_2_), and (**G**–**I**) to the effects of X_1_, X_2_ and X_3_ on the polydispersity index (Y_3_).

The systematic development of nanostructured formulations has gained momentum in recent decades through the use of design of experiments. This approach uses multivariate statistics and response surface modeling to obtain formulations with optimized characteristics with reduced resources of time, work and money [9,22,39,40]. The Box–Behnken design (BBD) is one of the main methodologies and is very popular in industrial research, because it is an economical design that requires only three levels for each factor. In this way, when analyzing three factors and three different variables, it has the advantage of requiring only 15 experiments [9,20,41,42]. Table 2 summarizes the data for the independent variables—the DOPE/CHEMS ratio (X_1_), docetaxel/lipid ratio (X_2_) and sonication time (X_3_)—related to encapsulation efficiency (Y_1_) particle size (Y_2_) and index of polydispersity (PDI) (Y_3_). A response surface methodology was used to verify the effects of variations in these parameters, and to determine the best preparation condition. It is possible to observe great variation in the three analyzed responses: encapsulation efficiency (13.45–89.36%), particle size (114.1–292.9 nm) and PDI (0.200–0.480). These variations may be associated with the complex effects of each of the independent factors and interactions with each other. For better observation and understanding of these effects, surface response surface graphs were generated (Figure 1).

**Figure 2:**
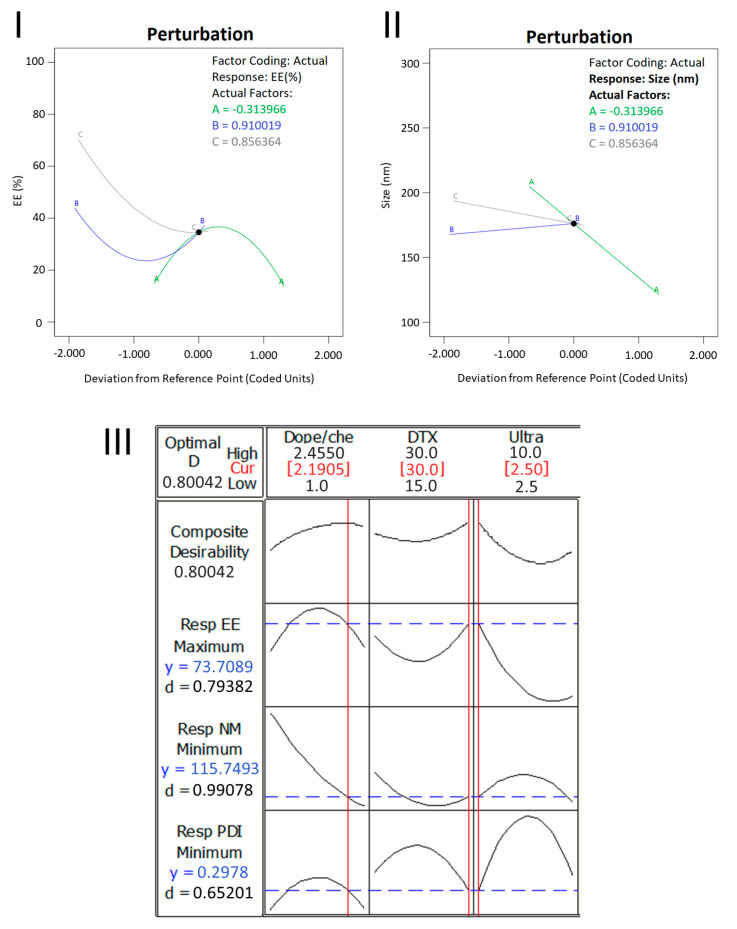
Perturbation plot and optimization graph. The perturbation plot (**I**) shows the effects of the variables DOPE/CHEMS ratio (green), docetaxel/lipid ratio (blue) and sonication time (gray) for encapsulation efficiency (EE%). Graph (**II**) shows the effects of the same variables on particle size (nm). Graph (**III**) presents the graphical optimization for the desired formulation.

Regarding the encapsulation efficiency responses and the effects attributed to independent factors shown in Figure 1A–C, the most striking influences observed were lipids proportions (DOPE and CHEMS) and molar ratio between docetaxel and lipids. In previous studies involving the development of liposomes, the proportions between two components and concentration of the drug in relation to lipids are two of the main factors most cited as critical for manufacturing, and consequently, developing new formulations [22]. The analysis of the perturbation plot in Figure 2I helps to compare the effects of all factors on encapsulation efficiency. The perturbation plot is used to compare the influences of factors in the common design space. A steep slope obtained for a variable shows that the parameter is sensitive to that factor [43,44]. In Figure 2I, it is possible to see a quadratic relationship of the variable effect X_1_ (DOPE/CHEMS ratio) on the encapsulation efficiency, and an opposite relationship of the variables X_2_ (docetaxel/lipids ratio) and X_3_ (sonication time) in relation to X_1_. The steep slope obtained by the sonication time denotes a negative effect and that the encapsulation efficiency is more sensitive to this factor. Heterogeneous variation of encapsulation efficiency, observed in Figure 1A–C and Table 2, is related to several factors causing different effects when observing the dependent factors. A higher ratio of docetaxel to lipids seems to promote greater vesicle organization, leading to smaller particle sizes, but with slightly lower encapsulation efficiency. The association between vesicle stabilization effects promoted by CHEMS and the drug interactions (of lipophilic nature) with the hydrocarbon chain seems to explain these influences. In a study using DOPE:CHEMS:DSPE-PEG 2000 for paclitaxel delivery, an encapsulation percentage of 90% was obtained, similar to two of our results (89.36% and 88.37%) [45].

Particle size is a critical quality attribute (CQA) often related to biodistribution, intracellular uptake capacity, encapsulation efficiency and stability [22,46,47]. To achieve the permeability and retention effect (EPR), the ideal size for nanoparticles is from 70 to 200 nm [45,48,49]. Liposome size also has an effect on formulation stability. Sizes smaller than 200 nm are more stable, due to the difference in lipid packaging that determines surface pressure in the lipid bilayer [45]. From Figure 1D–F, it is noted that the interaction between the independent variables X_1_ (DOPE:CHEMS ratio) and X_2_ (docetaxel/lipids ratio) causes a significant effect on particle size. Considering all interactions estimated in the conditions addressed, 80% of formulations presented in the present study are within the size range described in previous works as ideal (between 70 and 200 nm). The perturbation plot in Figure 2II shows a negative effect with a steep slope for the variable X_1_ (DOPE:CHEMS ratio) on particle size. The sonication time (X_3_) also has a negative effect, however, with a lower slope, and the variable X_2_ (docetaxel/lipids ratio) has a positive effect on particle size. Despite showing an opposed effect or additive impact, the results of the effects may not be statistically significant. In a previous study, when varying amplitude, cavitation cycle and time, a significant change was observed in the formation of unilamellar liposomes, and an amplitude of 40% was considered effective for obtaining nanometric sizes [32].

The polydispersion index (PDI) refers to a representation of the distribution of particle size populations in a given sample and PDI reference values can vary from 0.0 (for a perfectly homogeneous sample with respect to particle size) to 1.0 (representing a highly polydispersed and heterogeneous sample with diverse particle size populations). Of the fifteen experiments carried out, eleven showed polydispersion greater than 0.3. Previous studies describe values lower than 0.3 as ideal; however, values equal to or lower than 0.4 are considered acceptable for a liposome formulation [47,50,51,52]. By observing the effect of the independent variables and their interactions on polydispersity in Figure 1G–I, together with the data in Table 2, we noticed that only three values presented results greater than 0.4 for PDI. However, three results showed PDI values lower than 0.3, two of which (0.200 and 0.253) correlated with the tests with the best encapsulation efficiency results and the smallest observed particle size. The perturbation graph obtained for the evaluation of the PDI showed no effects of the variables on this parameter. Although the effects of the variables on the PDI were relatively discrete, the interactions between the three factors emphasized coherence between the analyzed data, favoring a good distribution of sample populations and low particle size [32].

An important parameter in evaluating stability of a colloidal system is zeta potential. Particles with high negative or positive zeta potential repel each other, indicating a stable system, whereas near-neutral values lead to liposomal aggregation. In addition, the nanosystem charge influences the time of systemic circulation and interactions with target tissue and may alter the opsonization profile of particles and the general profile of plasma circulation. Herein, the zeta potential values ranged from –18.8 mV to 0.15 mV. Previous studies, in liposomes of DOPE:CHEMS:DSPE-PEG 2000 composition, showed values close to neutrality, which can be attributed to DSPE-PEG 2000, considering that pegylation has a reducing effect on the electrostatic attraction between DOPE and H^+^ ions [45,53,54,55,56].

The responses Y_1_, Y_2_ and Y_3_—encapsulation efficiency, particle size and PDI, respectively—were subject to multiple regression, generating a second order polynomial equation in a full quadratic model, in which the highest reliability coefficients (R^2^) were selected based on linear equations [57]. The coefficient values and equations obtained for the model are described in Table 3.

The generated polynomial equations make it possible to study the effects of each factor and their interactions observed in the dependent factors considered. The magnitude of the coefficients and mathematical sign provide data on synergistic effects (positive charge) or antagonistic effects (negative charge). Based on the values obtained, it can be affirmed that there was a satisfactory adaptation of the regression for the encapsulation efficiency (Y_1_) and particle size (Y_2_) response model, as these presented the highest correlation coefficients (R^2^) [58].

For the encapsulation efficiency (Y_1_) the isolated term X_1_ and the interactions X_1_X_2_ and X_1_X_3_ favored the encapsulation efficiency response, although the isolated terms of X_2_ and X_3_ and their interaction (X_2_X_3_) had an antagonistic effect, negatively influencing the response. In the particle size response (Y_2_) the isolated terms DOPE/CHEMS molar ratio (X_1_) and sonication time (X_3_) and the interaction between them (X_1_X_3_) presented positive coefficients, denoting a favorable effect in obtaining the desired response. In the coefficients that presented the term X_2_ (molar ratio DTX/lipids) and in the interactions X_1_X_2_ and X_2_X_3_, the response was unfavorable due to negative coefficients, representing an antagonistic effect on these factors for the Y_2_ response [57]. The term corresponding to the molar ratio of lipids had statistical significance for the dependent factor Y_1_ (encapsulation efficiency). This result was expected, since the proportion of two lipid components is attributed as a critical point for the manufacture of liposomes with a potential to favor the Y_1_ response [22,57]. The other terms showed little statistical significance.

**Figure 3:**
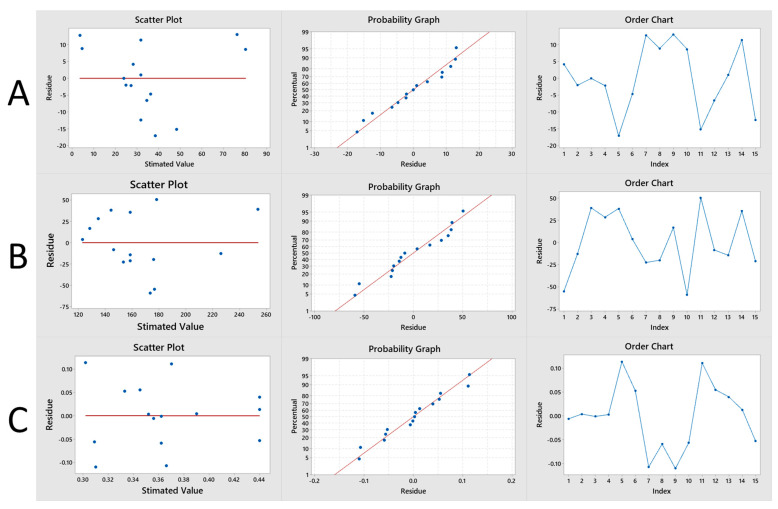
Graphs of normal probability (percentage vs. residual), of residuals versus adjusted values and observation order versus residual in relation to variables Y_1_, Y_2_ and Y_3_. The graphs were combined and aligned in the figure in relation to each response analyzed in the experimental design, in which the line graphs (**A**) relate to the encapsulation efficiency (Y_1_), in (**B**) to the particle size (Y_2_) and in (**C**) the polydispersity index (Y_3_).

Figure 3 presents the graphs of normal probability (percentage vs. residual), of residuals vs. adjusted values and of order of observation vs. residual. All were combined in the figure for each response analyzed in the experimental design. The responses from the normal probability plots denoted a normal distribution of estimated vs. experimental values. Considering the residuals vs. adjusted values plots, the PDI dependent factor plot (Y_3_) (Figure 3C) corresponds to the best attribution of homoscedasticity compared to the other responses [9,59]. The observation order vs. residual graphs, as well as the normal probability of residuals, showed acceptable residuals that supported the continuation of the analyses for formulation optimized by the statistical model [59].

During the project development study, desirable objectives were outlined, such as minimum particle size, minimum PDI and maximum encapsulation efficiency. Based on the experimental responses, a formulation optimized by the desirability criteria was selected [21,42]. The ideal formulation (Optimal-D) was generated according to the graph in Figure 2III, with desirability corresponding to 0.80042. The optimal conditions determined for the optimized formulation were a DOPE:CHEMS ratio of 2.2, a DTX:lipid ratio of 1:30 and a sonication time of 2.5 min. Under these conditions, the model established target values of 89.36% for encapsulation efficiency, 114.10 nm for particle size and 0.2 for PDI. The results obtained experimentally for optimized formulation, although slightly different from the values established by the model, were better than expected and within the previously targeted ideal ranges. The results predicted by the model for Y_1_, Y_2_ and Y_3_ were 73.7%, 115.7 nm and 0.297, respectively. The experimental values for optimized formulation were 88.65% of encapsulation efficiency (relative error = 20.3%), a particle size of 107.17 nm (relative error = 7.4%) and a polydispersity index equal to 0.213 (relative error 28.4%). 

**Figure 4:**
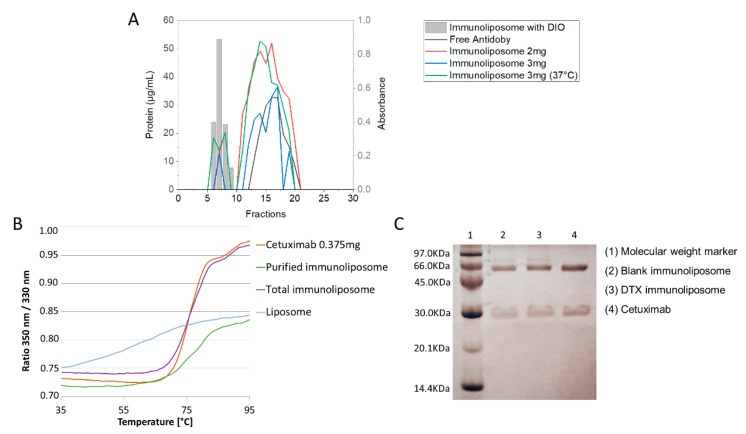
Liposome elution profile with DIO fluorophore, free antibody, docetaxel immunoliposome with 2 mg antibody, docetaxel immunoliposome with 3 mg antibody and docetaxel immunoliposome with 3 mg antibody (Incubation 37 **°**C) by size exclusion chromatography on Sepharose CL-4B (**A**) Graph of thermophoresis of cetuximab antibody, total immunoliposome, purified immunoliposome and liposome (**B**) SDS-Page gel electrophoresis for free cetuximab and blank and docetaxel-containing immunoliposomes (**C**).

For functionalization of the optimized liposome with the anti-EGFR antibody (cetuximab) following the direct conjugation method previously described, three different formulations were tested: with 2 mg and 3 mg of antibody incubated at room temperature and with 3 mg of antibody incubated at 37 °C for 2 h. In Figure 4A, it is possible to observe the elution profile in the CL-4B column of the two components of immunoliposome separately: liposome, which was labeled with fluorophore Dio, eluting in fractions 6–9, and cetuximab antibody in the unconjugated form, eluting in fractions 11 to 20. Thus, from these chromatograms, it was possible to distinguish the peaks of the chromatograms of the immunoliposome formulations. In the chromatograms shown in Figure 4A, it is possible to observe the separation of two peaks: the first referring to immunoliposomes (compound of higher molecular weight, in the same elution range as liposomes) and the second referring to antibodies that were not conjugated.

Table 4 shows the data of particle size, PDI, zeta potential and conjugation efficiency of the tested formulations. Based on the results presented, it is observed that the immunoliposome with 3 mg of conjugated antibody for 2 h at 37 °C showed higher conjugation efficiency (14.06%) when compared to the other formulations. Furthermore, it appears that functionalization did not significantly affect the physicochemical characteristics of liposome used for conjugation, retaining high rate of drug encapsulation. Regarding the nanoparticle size, an increase of approximately 50 nm in diameter was observed when compared to liposomes, possibly due to additional antibody on immunoliposome surface. However, it maintained the ideal size, less than 200 nm for penetration into solid tumors.

Previous studies also functionalized liposomes with a cetuximab antibody; however, lipid composition studied showed conjugation efficiency above 50%, possibly due to changes in the formulations [11,25,60]. Thus, it is believed that lipid DOPE present herein may be influencing the process of antibody conjugation onto the liposome surface. In an aqueous medium and at room temperature, DOPE molecules are not able to assume a bilayer structure, adopting an inverted hexagonal shape. In addition, DOPE has a small polar head, which helps in the tetrahedral molecular geometry and consequent intermolecular interactions between the amino and phosphate groups, explaining the tendency of these molecules to assume an inverted hexagonal shape. In the liposome, CHEMS molecules are sandwiched between DOPE molecules, inducing electrostatic repulsion between the amino and phosphate groups, further the formation of bilayer structures. On the other hand, the lipid phosphatidylcholine from soy (SPC), also widely used in liposome formulations, has a polar portion and hydrophobic chain with similar volume, presenting organization in cylindrical geometry and formation of a lamellar phase, being more stable to external interactions, such as for the conjugation of Mabs, providing a high conjugation efficiency, preserving the liposomal nanometric size and antibody integrity [11,45].

In addition, Jain et al. (2021) developed pH-sensitive liposomes functionalized with VEGF antibody for DTX encapsulation. The formulation consisted of DOPE:Chol:CHEMS:Lecithin, and the authors argued that, at neutral pH, the DOPE lipid acquires a hexagonal structure. However, when the CHEMS stabilizer is added, it forms a lipid bilayer, which, when in contact with acidic pH, slightly promotes the conversion of the lamellar layer into hexagonal. In addition, the increase in the amount of DOPE in the formulation, despite increasing the sensitivity at pH, reduces the percentage of encapsulation of the drug, probably due to the structural flexibility of DOPE and the low transition temperature. This last characteristic was reversed in the study through the increase in the amount of lecithin, which presents a higher phase transition temperature compared to DOPE, which improved encapsulation efficiency [61]. 

Regarding the incubation temperature for the functionalization reaction, Mamot et al. (2004) demonstrated that functionalization of cetuximab increased from 30–50% when incubated for 12 h at 37 °C, to 70–80% after incubation at 55 °C for 30 min [62]. Therefore, increasing the incubation temperature contributed to the enhancement in conjugation efficiency when compared to incubation at room temperature

According to SDS-Page gel electrophoresis bands shown in Figure 4C, it is observed that the process of conjugation with liposomes did not compromise the integrity of primary structure of cetuximab antibody, and in addition, the result confirms the presence of antibody in the formulation. Previous studies using the antibody cetuximab have already shown that under reducing conditions, two antibody bands can be observed: 50 kDa (heavy chain) and 25 kDa (light chain), which is in agreement with the result obtained in the figure below [11].

Microscale thermophoresis or thermal diffusion is a first-choice technique for studying biomolecular interactions, and has been widely used to study binding, labeling specificity and protein stability [63]. Figure 4B shows the comparative thermal stability assay profiles for cetuximab and samples (purified immunoliposome, total immunoliposome and liposome). As the system is heated, the fluorescence signal indicates a change in protein folding: this change or transition is called the inflection temperature. In Figure 4B, the monoclonal antibody cetuximab presents a curve with a more accentuated displacement, indicating inflection temperatures of 75.6 °C and 89.7 °C, especially because it is the complete antibody molecule and presents high fluorescence due to high frequency of residues in the protein sequence. The liposome presented inflection temperatures of 51 °C and 65 °C, while the purified immunoliposome samples presented 51.5 °C, 73.8 °C and 80.1 °C and the total immunoliposome presented 75.3 °C and 89.3 °C. It became evident that the inflection points of the isolated samples (liposome and cetuximab) were repeated in the more complex samples (total immunoliposome and purified immunoliposome); that is, the liposome inflection temperatures of 51 °C were repeated for purified immunoliposome. However, it was not so evident in the total immunoliposome sample. Cetuximab inflection points were maintained in all other immunoliposome samples with very close values, which can evidence the conjugation of the antibody to the nanoparticle and maintain the structure and stability of antibody, guaranteeing its activity. 

**Figure 5:**
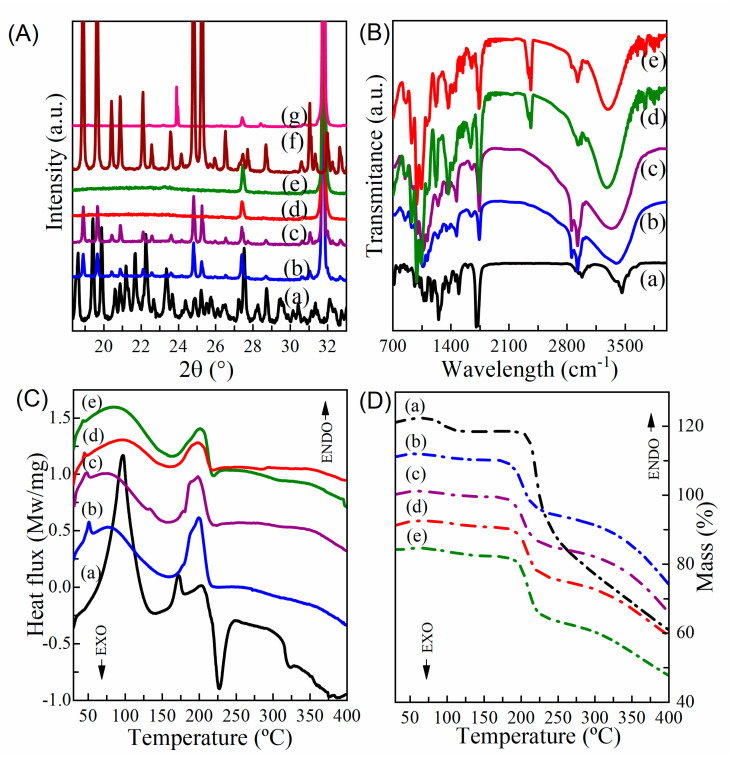
Solid state characterization of lyophilized liposomes: (**A**) powder X-ray: diffraction patterns of DTX (**a**), unloaded liposome (**b**), liposome DTX (**c**), unloaded immunoliposome (**d**), DTX immunoliposome (**e**), PBS (**f**) and sucrose (**g**). (**B**) FTIR: DTX (**a**), unloaded liposome (**b**), liposome DTX (**c**), Cetuximab (**d**), unloaded immunoliposome (**e**). (**C**) Differential scanning calorimetry: curves of DTX (**a**), unloaded liposome (**b**), DTX liposome (**c**), unloaded immunoliposome (**d**) and DTX immunoliposome (**e**). (**D**) Thermogravimetry: curve of DTX (**a**), unloaded liposome (**b**), DTX liposome (**c**), unloaded immunoliposome (**d**) and DTX immunoliposome (**e**). The curves were vertically translated for clarity.

Liposomes and immunoliposomes were characterized by Fourier transform infrared spectroscopy (FTIR). FTIR analysis makes it possible to identify the presence of chemical groups in the drug and carrier and evaluate the intermolecular interactions between different compounds through changes in spectral peaks corresponding to specific chemical groups. In Figure 5B, docetaxel presents the main peak represented by the vibration of the C=O ester bond at approximately 1722 cm^−1^. It is observed that the bands at 1165 cm^−1^ e 1247 cm^−1^ show the characteristic C-O stretching of ester [11]. The spectrum shows the peak referring to the N-H stretching of secondary amide (band in 3460 cm^−1^). It is also observed the strong stretching of C=O at 1701 cm^−1^, characteristic of amide. The spectrum also revealed bands at 1490 cm^−1^, 1452 cm^−1^ consistent with the C=C aromatic ring. In the spectrum of the blank liposome, a characteristic main peak can be observed at 1740 cm^−1^ (COOH vibration), as well as a wide O-H stretch between 3180–3500 cm^−1^. The peaks at 2853/2923 cm^−1^ can be related to the symmetrical and asymmetrical CH2 stretching of the DSPE-PEG 2000 structure [64]. Weak absorption at 1645 cm^−1^ indicates C=C stretching and the C-O-C stretching (1059 cm^−1^ and 995 cm^−1^) belongs to the DSPE-PEG portion. The asymmetric stretching vibration of the P=O group can be identified by a peak at 1238 cm^−1^, which can be attributed to DSPE or DOPE, phospholipids. As for the liposome containing DTX (Figure 5B), the characteristic peaks of the drug (N-H stretch of the amide at 3460 cm^−1^ and vibration of the ester bond around 1722 cm^−1^) are not in evidence, which could be explained by the fact that the drug is encapsulated in the matrix structure of the liposome, or due to the overlapping of the chemical bond bands of DTX and lipids [11]. Figure 5B(d,e) shows the FTIR spectra of the free cetuximab and blank immunoliposome, in order to evaluate the antibody functional groups and their presence in the immunoliposome structure after functionalization. Cetuximab exhibits a characteristic peak identified at 3270 cm^−1^ in Figure 5B, which corresponds to the stretching vibration due to the O-H groups. Additionally, 2931 cm^−1^ is the vibration attributed to the asymmetric stretching of C-H. Another characteristic peak is evidenced at 1696 cm^−1^, characteristic of the N-H group, and at 1712 cm^−1^, attributed to the C=O stretching of amide [65]. The characteristic peaks of the antibody were also found in the spectrum of the blank immunoliposome, signaling that cetuximab was attached to the nanoparticle with the appearance of the characteristic chemical groups. These peaks are more subtle, because the antibody is diluted in the formulation.

The encapsulation of the nanoparticles was monitored through PXRD analysis (Figure 5A). The diffraction pattern of the pure drug is in a good agreement with the crystalline structure reported by Vella-Zarb and coworkers (2013) [66]. It is not possible to observe any peaks derived from DTX in any of the liposomes and immunoliposomes. Furthermore, the loaded and unloaded liposome, as well as the loaded and unloaded immunoliposome, exhibited remaining peaks from PBS, used in the production process, while the first two mentioned also presented peaks related to sucrose, used in the lyophilization process, suggesting that the optimized formulation is semi crystalline in nature. 

The thermal analysis was carried out in order to monitor the thermal behavior of the drug-loaded and precursor compounds. In this sense, DSC and TG curves (Figure 5C,D) were recorded, as the physical state can influence drug release [67]. It is possible to realize that the pure drug presents an endothermic peak at 171 °C, which is in agreement with the melting point reported in the literature [68], as well as an exothermic event at 216.4 °C, related to the decomposition process. The liposome and immunoliposome exhibited events below 100 °C that corresponds with the release of water, and it is possible to notice a sharp drop in mass in thermogravimetry, related to their decomposition process. It is interesting to point out that the decomposition events of PBS and sucrose occur at a higher temperature [69,70], and therefore, these events were not noticeable in the TG of the loaded and unloaded liposomes and immunoliposomes.

**Figure 6:**
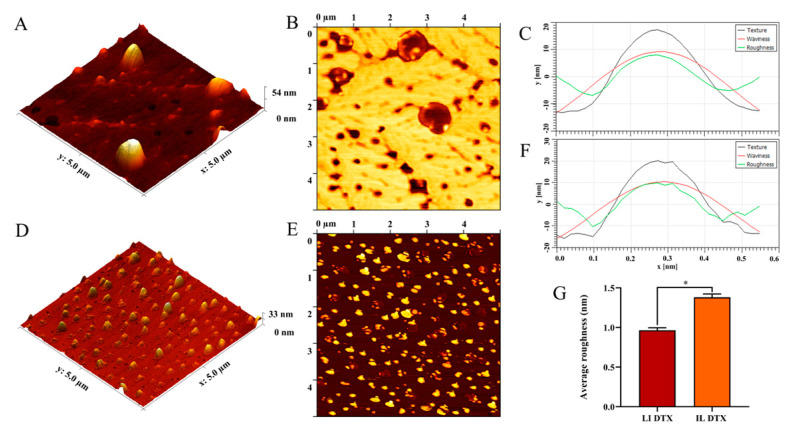
Image of the morphology of the liposome with drug (**A**,**B**), immunoliposome with drug (**D**,**E**), histograms of roughness of the liposome with drug (**C**), histograms of the roughness of the immunoliposome with drug (**F**), graph of mean roughness of liposome and immunoliposome DTX (**G**). * *p* < 0.05.

The liposomes had homogeneous distribution and spherical morphology, as found by Takechi-Haraya et al. (2020) who encapsulated calcein in liposomes composed of egg phosphatidylcholine and cholesterol [71]. Figure 6B shows lipid membrane stains derived from liposome disruption, probably derived from liposomes with higher substrate adhesion energy. The surface of individual liposomes is smooth (Figure 6) and when cetuximab was bound to liposomes, small globular structures, located on the liposomal surface became visible, exhibiting the highest rate of appearance (Figure 6). The roughness histograms can be seen in Figure 6C-D, the black line represents the texture of the liposome contour, while the red line represents the undulations. The sum of the peaks and valleys of the ripples was performed and a mean line was drawn showing the roughness (green line). As can be seen, there was a change in the surface roughness profile of the immunoliposome (Figure 6C) in relation to the liposome (Figure 6D). The same behavior was observed in the average roughness values (*Ra*) (Figure 6E). Due to the presence of the antibody on its surface, the DTX immunoliposome had its *Ra* significantly increased to 1.382 ± 0.041 nm, in relation to the DTX liposome, which presented *Ra* of 0.965 ± 0.0322 nm [72].

**Figure 7:**
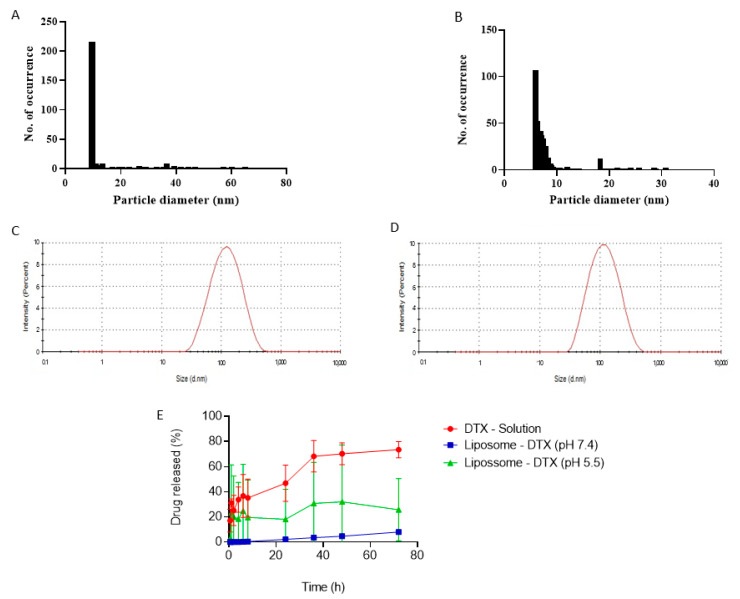
Particle size distribution: DTX liposome by MFA (**A**), DTX liposome by DLS (**C**), DTX immunoliposome by MFA (**B**), DTX immunoliposome by DLS (**D**), in vitro release study of DTX-loaded liposome and DTX solution using dialysis membrane (**E**).

Figure 7A–D shows a comparison of the liposome size distribution plots by AFM and DLS, and it can be seen that the liposomes with DTX have a more homogeneous size distribution compared to the immunoliposome with DTX. It became evident that the liposome sizes by AFM were smaller, where the liposomes with DTX had an average size of 19.54 ± 1.498 nm and the immunoliposome with DTX an average size of 14.63 ± 0.9040 nm. Compared to the particle size by DLS, liposomes with DTX and immunoliposomes were 107.2 ± 2.9 nm and 156.77 ± 1.67 nm, respectively. This difference is justified because the DLS technique evaluates the hydrodynamic diameter, while the AFM shows the actual size of the nanostructures [73].

The results shown in Figure 7E shows that liposomes in citrate buffer pH 5.5 have an increased DTX release profile when compared to the release profile of liposomes in phosphate buffer pH 7.4. At the end of 72 h, the formulation at pH 7.4 releases about 10.45%, while at pH 5.5 it releases a little more than 25%, demonstrating that the liposome is pH-responsive, potentially releasing more drug in the acidic tumor environment [74,75]. Figure 7E also shows higher in vitro DTX release for the commercial formulation, a docetaxel solution in a vehicle composed of dehydrated alcohol and polysorbate 80. Liposomes released less DTX because they act as controlled release systems, able to accumulate in the tumor by the EPR effect.

**Figure 8:**
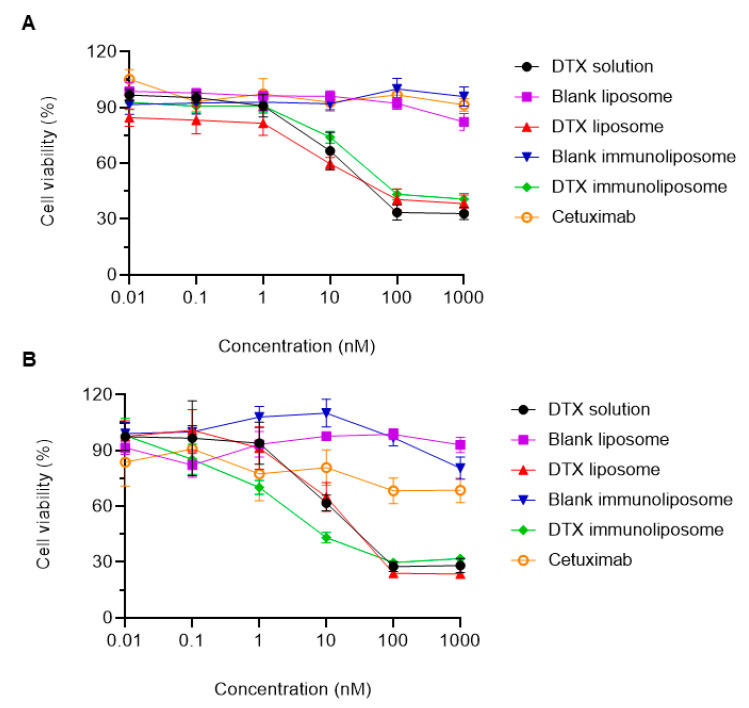
Cell viability by the MTT assay at 72 h incubation. PC3 cell line (**A**), DU145 cell line (**B**).

Figure 8A,B shows the cell viability of the two cell lines used (PC3 and DU145), with the following samples tested: DTX solution, blank liposome, DTX liposome, blank immunoliposome, DTX immunoliposome and cetuximab. Our hypothesis is that the functionalization of the liposome with cetuximab will provide a better targeting of cells that overexpress the EGFR receptors (DU145), consequently acting to improve the cytotoxic effect of the immunoliposome in this cell line. In the PC3 cell (Figure 8A), it is observed that the formulations showed a greater cytotoxic effect from the concentration of 10 nM, with 33.36% for DTX solution, 40.26% for LIP-DTX and 25.96% for IL -DTX. As expected, due to the lower amount of EGFR receptors, IL-DTX showed less toxicity when compared to the other treatments. Note that, at the lowest concentration tested (0.01 nM), LIP-DTX already showed a greater toxic effect (15.14%) than the DTX solution (7.24%). A concentration-dependent relationship was also observed in the three formulations with DTX, that is, by increasing the concentration of the treatment groups, the cytotoxic effect increases in the same way. Regarding the DU145 cell (Figure 8B), greater toxicity was also observed at a concentration of 10 nM, with a similar toxic effect between LIP-DTX (34.92%) and DTX (38.28%). However, IL-DTX showed a toxic effect from the concentration 0.1 nM (14.78%), appearing more pronounced in the other concentrations: 1 nM (29.86%), 10 nM (56.86%), 100 nM (70.28%) and 1000 nM (68.18%). Thus, this result was possibly due to the greater expression of EGFR receptors in this cell, showing greater affinity for the anti-EGFR antibody (cetuximab) present on the surface of the immunoliposome. It was also noted that in both cell lines, the blank liposome, blank immunoliposome and cetuximab did not have a relevant cytotoxic effect, demonstrating that lipids and free antibodies have no effect on the reduction in cell viability observed by IL-DTX.

Eloy et al. (2020) evaluated the in vitro cytotoxic effect of liposomes of lipid composition SPC:Chol:DSPE-PEG conjugated with cetuximab, demonstrating that the immunoliposome loaded with DTX was more cytotoxic than DTX in solution and DTX encapsulated in liposomes in the DU145 cell, due to increased EGFR expression. For the DU145 cell, the pH-sensitive immunoliposome, at a concentration of 100 nM led to cell viability of 29.72 ± 0.88%, while the conventional immunoliposome in the study by Eloy et al., (2020) showed viability of 40.58 ± 6.99%. Such a difference was also observed in PC3 cells and with the pH sensitive liposome in both cell lines, possibly due to the pH sensitivity of the formulation, which leads to higher cytotoxicity [11]. Sicard et al. (2020) developed immunoliposomes containing HER2-targeted antisense oligonucleotides (ASO) and tested using in vitro models of 2D and 3D spheroids. Encapsulated ASO was more effective than free ASO, with differences being observed at 4 and 24 h. At 4 h, a short exposure time, ASO liposomes were more effective than ASO immunoliposomes. However, at 24 h, a long exposure time, the immunoliposome performed better than the liposome in PC3 cells [76].

From Table 5, it was verified that both samples, LIP-DTX and DTX solution, presented lower IC_50_ for DU145 (28.28 and 33.55 nM, respectively) when compared to PC3 (65.74 and 55.77 nM, respectively), thus being more cytotoxic for that cell line. Furthermore, in relation to the immunoliposome, as expected, the lowest IC_50_ among all formulations was observed for DU145 (12.60 nM), demonstrating, when compared to the IC_50_ of DTX solution (33.55 nM), that there was a greater targeting of the nanoparticle to this cell line, due to the higher expression of anti-EGFR receptors. Therefore, the immunoliposome increased cytotoxicity, with a decrease in IC_50_, which is equivalent to a value of 2.66 and 2.24 times lower than the DTX solution and the liposome, respectively, in DU145 cells.

**Figure 9:**
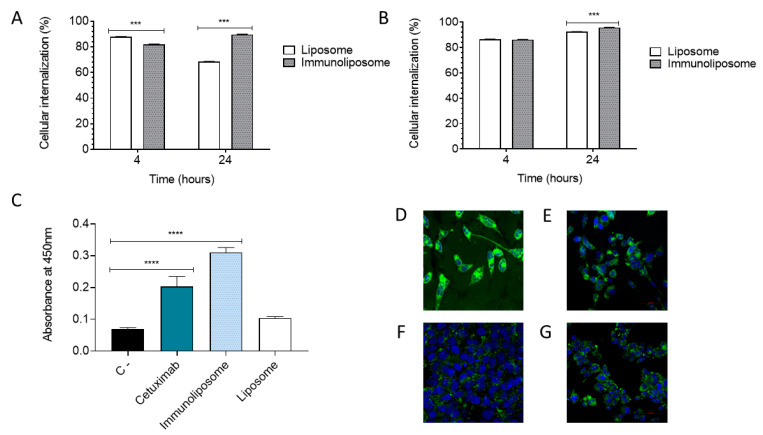
Cellular internalization of the liposome and immunoliposome. PC3 cell line (**A**), DU145 cell line (**B**). Indirect ELISA to assess the binding ability of cetuximab, immunoliposome and liposome to the EGFR antigen (**C**). Confocal microscopy (20× objective) of liposome in PC3 cells (**D**), immunoliposome in PC3 cells (**E**), liposome in DU145 cells (**F**), immunoliposome in DU145 cells (**G**). Two-way ANOVA test with Bonferroni posttest between samples. *** *p* < 0.01 (**A**,**B**). One-way ANOVA test with Tukey posttest between samples. **** *p* < 0.05 (**C**).

The indirect ELISA assay suggests that the cetuximab was successfully conjugated to the liposome in the correct conformation to keep its functionality of binding to EGFR. It is important to point out that this result supports the finding related to the uptake cellular because after coupling the antibody to the liposome, it remains functional, therefore improving the uptake cellular when compared to the non-functionalized liposome. In a study conducted by Mack and colleagues (2012) a dual-targeted immunoliposome was developed to recognize two antigens EGFR and CEA. With the ELISA assay, the bispecific immunoliposome showed the ability to bind for both antigens, corroborating the findings related to the capacity of the immunoliposome to bind a range of cancer cell lines expressing EGFR and/or CEA [77]. Figure 9C shows that the immunoliposome had a higher absorbance when compared to cetuximab, probably due to the presence of conjugated and non-conjugated antibodies, increasing the observed signal. It is notable that the liposome, as expected, showed similar absorbance to the negative control, disregarding the interference of lipids in binding affinity.

In treatment of cancer, monoclonal antibodies can exert their therapeutic effect by direct mechanisms (blocking cell signaling) or by indirect mechanisms (activation and regulation of the immune system). In this study, the cetuximab antibody present in EGFR-targeted immunoliposomes was designed to function for targeting, binding to receptors present on the surface of cancer cells, which can be demonstrated by cell internalization studies. Additionally, taxane nanoparticles have demonstrated the ability to provide immune-regulation resulting in immunogenic cell death [78,79,80]. 

Figure 9A,B show the flow cytometry studies in the two cell lines (PC3 and DU145) and with two groups of tested formulations (liposome and immunoliposome). The times of 4 h and 24 h of treatment were chosen for analysis. In the 4 h period, for PC3, the liposome presented 86.64% (Figure 9A) of cellular uptake in living cells, while the immunoliposome presented 81.47% (Figure 9A). In the DU145 cells, a similar uptake was observed for the liposome with 86.32% (Figure 9B); however, for the immunoliposome, when compared to PC3 (81.47%), there was a higher percentage of cell uptake at 86.10% (Figure 9B). Therefore, in the PC3 cell line, with low EGFR expression, the immunoliposome does not show greater cellular uptake than the liposome, as expected. Comparatively, at 24 h (Figure 9A,B), a greater cellular uptake of the immunoliposome was also observed in the DU145 cell line (Figure 9B), with 96.54% of internalization, when compared to the immunoliposome in the PC3 cell line (Figure 9A), with 89.41%, showing significant statistical difference. This result corroborates our hypothesis, demonstrating that by having more EGFR receptors, the DU145 cell line is able to better internalize the immunoliposome. In addition, these results also corroborate the internalization of liposomes and immunoliposomes observed in confocal microscopy after a period of 24 h (Figure 9D–G).

In agreement with our study, Petrilli et al., (2018) evaluated the cellular internalization of anti-EGFR immunoliposomes by flow cytometry in skin cancer cells A431 (EGFR positive) and B16F10 (EGFR negative). In the A431 cell line, an approximately three-fold increase in internalization was observed when the cells were treated with the immunoliposome. As for the B16F10 cells, the uptake was similar between liposomes and immunoliposomes, since there is no EGFR expression for binding of cetuximab present on the immunoliposome surface. Such results were also corroborated by confocal microscopy [25].

## 4. Conclusions

Using the design of experiments strategy, it was possible to obtain an optimized formulation (DOPE/CHEMS ratio of 2.2; docetaxel:lipid ratio of 1:30 and sonication time of 2.5 min) with high encapsulation efficiency, low polydispersity and nanometric particle size. Cetuximab conjugation onto the liposomal surface was not high, but did not disturb the liposomal physicochemical characteristics. We were able to demonstrate the success of the Mab conjugation by a variety of techniques, including gel filtration chromatography, electrophoresis and thermophoresis. Solid state characterizations evidenced successful drug encapsulation, with reduced crystallinity. DTX release was higher under acidic pH. It was possible to evidence the high extent of EGFR recognition of DU145 prostate cancer cells by the immunoliposome. The immunoliposome showed greater cytotoxicity in the DU145 cell line, so the functionalization process ensured an increase in the cytotoxicity of the nanoparticle, which can be explained by the high expression of EGFR. While in the PC3 cell line (low EGFR expression), the liposome was more cytotoxic. Regarding the internalization of nanoparticles, after 24 h, the immunoliposome was the most internalized in the two cells tested; however, in DU145, a higher percentage of the immunoliposome was captured by the cells compared to the PC3 cells. Future studies will investigate the effect of the formulations in prostate cancer xenografts in vivo.

## Figures and Tables

**Table 1:** Variables in the Box–Behnken design.

Independent Variable		Level Used		
	Symbol	Low (−1)	Medium (0)	High (+1)
X_1_: DOPE:CHEMS ratio	DP/CHEM	1	1.5	2.5
X_2_: Docetaxel:lipids ratio	DTX/LP	1:15	1:20	1:30
X_3_: Sonication time	ST	2.5 min	5 min	10 min
**Fixed Component**		DSPE-PEG 0.5 mol		
**Response variables**		**Goal**		
Y_1_: Encapsulation efficiency (%)	EE (%)	Maximum (100%)		
Y_2_: Particle size (nm)	Size (nm)	Optimum (<200 nm)		
Y_3_: Polydispersity index	PDI	Minimize		

**Table 2:** Box–Behnken design representing experimental runs with independent variables at three levels (−1, 0, 1) and observed responses (EE%, Size and PDI).

Formulation Run	X_1_ (DP/CHEM)		X_2_ (DTX/LP)		X_3_ (ST)		Y_1_ [EE (%)]	Y_2_ [Size (nm)]	Y_3_ (PDI)
	Level	Tested Ratio	Level	Tested Ratio	Level	Time Tested			
1	−1	1.0	−1	1:15	0	5	32.4 ± 2.28	121.6 ± 2.2	0.350 ± 0.019
2	1	2.5	−1	1:15	0	5	22.88 ± 1.12	213.3 ± 7.3	0.394 ± 0.032
3	−1	1.0	1	1:30	0	5	23.88 ± 4.89	292.9 ± 21.9	0.361 ± 0.044
4	1	2.5	1	1:30	0	5	25.15 ± 6.00	163.1 ± 0.8	0.355 ± 0.004
5	−1	1.0	0	1:20	−1	2.5	21.42 ± 2.08	182.5 ± 1.8	0.416 ± 0.005
6	1	2.5	0	1:20	−1	2.5	31.73 ± 3.21	126.8 ± 3.1	0.386 ± 0.037
7	−1	1.0	0	1:20	1	10	16.36 ± 3.70	130.9 ± 4.3	0.259 ± 0.001
8	1	2.5	0	1:20	1	10	13.45 ± 0.57	156 ± 0.4	0.303 ± 0.018
9	0	1.5	−1	1:15	−1	2.5	89.36 ± 8.77	145.1 ± 0.7	0.200 ± 0.004
10	0	1.5	1	1:30	−1	2.5	88.97 ± 13.78	114.1 ± 0.6	0.253 ± 0.009
11	0	1.5	−1	1:15	1	10	33.26 ± 9.17	228.8 ± 6.2	0.481 ± 0.047
12	0	1.5	1	1:30	1	10	28.01 ± 6.43	137.7 ± 1.2	0.400 ± 0.022
13	0	1.5	0	1:20	0	5	32.72 ± 6.85	144.0 ± 6.4	0.480 ± 0.013
14	0	1.5	0	1:20	0	5	43.17 ± 5.24	193.9 ± 27.2	0.453 ± 0.043
15	0	1.5	0	1:20	0	5	19.31 ± 0.74	137.1 ± 1.9	0.387 ± 0.198

**Table 3:** Regression analysis for the encapsulation efficiency (Y_1_), particle size (Y_2_) and polydispersity index (Y_3_) using an interaction model based on the effect of the DOPE:CHEMS ratio (X_1_), docetaxel:lipids ratio (X_2_) and sonication time (X_3_).

	Y_1_ [EE (%)]		Y_2_ [Size (nm)]		Y_3_ (PDI)	
	Coeff.	*p*-Value	Coeff.	*p*-Value	Coeff.	*p*-Value
**Intercept**	139.426	0.345	108.439	0.822	−0.545	0.580
**X_1_**	155.460	0.094	2.211	0.993	0.331	0.553
**X_2_**	−15.354	0.138	−5.294	0.866	0.039	0.545
**X_3_**	−19.119	0.178	41.911	0.362	0.089	0.336
**X_1_^2^**	−47.378	0.051	36.928	0.587	−0.076	0.581
**X_2_^2^**	0.346	0.109	0.480	0.466	−0.001	0.549
**X_3_^2^**	1.388	0.109	−2.482	0.355	−0.006	0.284
**X_1_X_2_**	0.304	0.843	−7.727	0.182	−0.002	0.847
**X_1_X_3_**	0.289	0.925	4.016	0.704	−0.003	0.882
**X_2_X_3_**	−0.159	0.597	−0.684	0.509	0.000	0.917
**R^2^**	81.81(%)		51.42(%)		30.66(%)	
**Regression equation of the fitted model**						
$Y_{1}=139.426+155.460X_{1}-15.345X_{2}-19.119X_{3}+0.304X_{1}X_{2}+0.289X_{1}X_{3}-0.159X_{2}X_{3}-47.378X_{1}^{2}+0.346X_{2}^{2}+1.388X_{3}^{3}$						
$Y_{2}=108.439+2.211X_{1}-5.294X_{2}+41.911X_{3}-7.727X_{1}X_{2}+4.016X_{1}X_{3}-0.684X_{2}X_{3}+36.928X_{1}^{2}+0.480X_{2}^{2}-2.482X_{3}^{3}$						
$Y_{3}=-0.545586+0.331X_{1}+0.039X_{2}+0.089X_{3}-0.002X_{1}X_{2}-0.003X_{1}X_{3}-0.0002X_{2}X_{3}-0.076X_{1}^{2}-0.001X_{2}^{2}-0.0059X_{3}^{3}$						

The interaction terms are represented by the association of factors (i.e., X_1_X_2_, X_1_X_3_ and X_2_X_3_) and the quadratic relationships are represented by higher order terms (i.e., X_1_^2^, X_2_^2^ and X_3_^2^). R^2^ represents the coefficient of determination.

**Table 4:** Physicochemical characterization of immunoliposome formulations prepared by direct conjugation.

**Immunoliposome with 2 mg Antibody**			
	Blank Immunoliposome *	DTX Immunoliposome *	
Size (nm)	107.4 ± 2.05	106.8 ± 4.45	
PDI	0.224 ± 0.013	0.224 ± 0.013	
Zeta (mV)	−16.7 ± 1.42	-17.1 ± 1.50	
EC%	9.84%	10.68%	
EE%	-	85.84 ± 3.70	
**Immunoliposome with 3 mg Antibody**			
	Blank immunoliposome *	DTX immunoliposome *	**DTX immunoliposome at 37 °C**
Size (nm)	111.07 ± 0.51	111.47 ± 0.49	156.77 ± 1.67
PDI	0.197 ± 0.01	0.245 ± 0.01	0.245 ± 0.00
Zeta (mV)	−17.77 ± 1.20	−17.77 ± 1.20	−17.67 ± 1.35
EC%	11.65%	6.91%	14.06%
EE%	-	75.8 ± 15.30	86.0 ± 14.29

* RT (room temperature).

**Table 5:** IC_50_ of the formulations tested in the two cell lines (PC3 and DU145) with their respective confidence interval.

**PC3**		
**Formulation**	**IC_50_ (nM)**	**Confidence interval**
DTX solution	55.77 ± 9.21	38.76 to 81.80
DTX liposome	65.74 ± 14.61	41.17 to 110.3
DTX immunoliposome	152.1 ± 25.43	106.2 to 225.5
Cetuximab	-	-
**DU145**		
**Formulation**	**IC_50_ (nM)**	**Confidence interval**
DTX solution	33.55 ± 7.20	20.91 to 55.33
DTX liposome	28.28 ± 4.60	19.80 to 40.99
DTX immunoliposome	12.60 ± 2.50	8.128 to 19.91
Cetuximab	-	-

## Data Availability

The data presented in this study are available on request from the corresponding author.
